# The costs of over-control in anorexia nervosa: evidence from fMRI and ecological momentary assessment

**DOI:** 10.1038/s41398-021-01405-8

**Published:** 2021-05-21

**Authors:** Sophie Pauligk, Maria Seidel, Sophia Fürtjes, Joseph A. King, Daniel Geisler, Inger Hellerhoff, Veit Roessner, Ulrike Schmidt, Thomas Goschke, Henrik Walter, Alexander Strobel, Stefan Ehrlich

**Affiliations:** 1 Division of Psychological and Social Medicine and Developmental Neuroscience, Translational Developmental Neuroscience Section, Faculty of Medicine, Technische Universität Dresden, Dresden, Germany; 2 Eating Disorder Research and Treatment Center, Department of Child and Adolescent Psychiatry, Faculty of Medicine, Technische Universität Dresden, Dresden, Germany; 3 Section of Eating Disorders, Department of Psychological Medicine, Institute of Psychiatry, Psychology & Neuroscience, King’s College London, London, UK; 4 Faculty of Psychology, Technische Universität Dresden, Dresden, Germany; 5 Department of Psychiatry and Psychotherapy, Charité Berlin, Berlin, Germany

**Keywords:** Psychiatric disorders, Human behaviour

## Abstract

A growing body of evidence suggests that a high level of self-control may, despite its positive effects, influence cognitive processing in an unfavorable manner. However, the affective costs of self-control have only rarely been investigated. Anorexia nervosa (AN) is an eating disorder that is often characterized by excessive self-control. Here, we used fMRI to explore whether over-control in AN may have negative affective consequences. 36 predominantly adolescent female AN patients and 36 age-matched healthy controls (HC) viewed negative and neutral pictures during two separate fMRI sessions before and after 10 min of rest. We tested whether abnormally elevated neural activity during the initial presentation in a brain region broadly implicated in top-down control, the dorsolateral prefrontal cortex (dlPFC), could predict subsequent activation in limbic areas relevant to bottom-up affective processing. Using ecological momentary assessment (EMA), we also tested for associations between the aforementioned neuroimaging markers and negative affective states in the two weeks following the experiment. fMRI data revealed that higher initial activation of the dlPFC in AN predicted increased amygdala reactivity during the second fMRI session, which in turn was related to increased self-reported tension during two weeks following the scan. These data suggest that over-control in AN patients may come at a cost including negative affective states on a short (minutes) as well as a longer time scale (days). This mechanism may significantly contribute to the persistence of AN.

## Introduction

Self-control, defined as the “ability to override or change one’s inner responses, as well as to interrupt undesired behavioral tendencies”^1^, is essential for physical health, mental well-being, interpersonal success and academic achievement^2^. Although generally desirable, self-control may be inherently costly and excessive self-control might be disadvantageous or harmful. Building on the notion that suppressing unwanted thoughts makes them, ironically, more likely to re-occur^3^ and influence behavior^4^, it has been proposed that self-control is a finite resource that becomes depleted by prior exertion^5^. After engaging in high levels of self-control (e.g., in attention control or emotion regulation (ER) tasks), participants show poorer performance in subsequent tasks that require control resources, such as response inhibition and error detection^6–10^. Neuroimaging studies have helped to understand the mechanisms underlying such costs of control^11,12^. In functional magnetic resonance imaging (fMRI), the decrement of behavioral performance that follows tasks requiring high self-control has been associated with decreased activation in the dorsolateral prefrontal cortex (dlPFC)^6,8,10^; a brain region broadly implicated in higher-order control of behavior, cognitive and affective processing^13,14^. In addition to the cognitive costs of exerting control, effects on affective processing (e.g., heightened amygdala reactivity) are well-documented^12,15^. The latter effects are especially relevant because affective processing is a critical prerequisite for psychological well-being that interacts with and modulates cognitive processing^16–18^.

A small number of fMRI studies have provided evidence suggesting that the exertion of self-control alters processing of aversive emotional stimuli. Wagner and Heatherton^19^ found that participants who performed an attention control task showed both increased amygdala activity and reduced functional connectivity between amygdala and ventromedial prefrontal cortex when subsequently viewing negative emotional scenes. Walter et al.^20^ described heightened amygdala activation as a paradox ‘rebound’ effect that occurred 2–3 s after the successful down-regulation of the amygdala response to negative pictures. Interestingly, the amplitude of this post-regulatory ‘rebound’ was positively correlated with self-reported habitual thought suppression and amygdala activation in response to the same stimuli when presented again in a passive viewing condition. The costs of control are also apparent when processing primary rewards. In chronic dieters, performing an effortful attention task subsequently led to greater food-cue related activity in a brain region involved in reward processing (orbitofrontal cortex) and was associated with reduced connectivity between this area and inferior frontal regions involved in inhibitory control^21^. This is in line with the observation that self-imposed inhibition of food intake leads to increased emotional responsiveness, depressive symptoms, and a strong preoccupation with thoughts about food^22,23^. In summary, it seems that the excessive or prolonged exertion of self-control may increase the reactivity of brain areas crucial to the processing of aversive and rewarding stimuli, while diminishing the capacity to recruit prefrontal areas involved in top-down control.

When studying the costs of (excessive) self-control, anorexia nervosa (AN) may be a disorder that can help to understand general psychological mechanisms^24^. Perfectionism and an excessive amount of self-control are central elements in theoretical models of AN (e.g., Polivy & Herman^25^, Fairburn et al.^26^, Haynos & Fruzzetti^27^, Kaye et al.^28^). Heightened self-control is closely related to core symptoms of the disorder, such as food restriction and excessive physical activity. It can also explain additional disorder-related behavior (e.g., calorie-counting, endurance of hunger and cold^29^) as well as typical but disorder-unrelated features (e.g., above average educational achievement^30^). It also seems to contribute to the high treatment resistance of patients with AN^31^. Furthermore, AN patients have been reported to display an increased capacity to delay rewards^32,33^ and high risk aversion^34,35^. As neural correlates of these behavioral patterns, acutely ill and recovered AN patients display an increased involvement of the fronto-parietal control network, especially the dlPFC, across a range of tasks employing disorder-related^36,37^ and disorder unrelated stimuli^38–41^. Individuals with AN have also been found to show increased functional connectivity in the fronto-parietal control network in the resting state^42,43^ and during the processing of rewarding^40^ and food stimuli^44^. There are discrepancies in the literature, however, as some task-based studies have also reported hypo-activation in lateral prefrontal regions in AN (e.g., Steward et al.^45^; Holsen et al.^46^).

To our knowledge, only one study has focused specifically on the costs of over-control in the domain of affective processing in AN. Seidel et al.^47^ reported a stronger volitional down-regulation of the brain reward system in response to positive pictures to be associated with higher body-related rumination, negative affect and tension (assessed in real-life via ecological momentary assessment, EMA) as well as deficient treatment response during follow-up. This may indicate that the tendency toward over-control in AN interferes with psychological well-being and, potentially, recovery. The aim of the current study was to further explore whether increased self-control in AN has costs in the domain of negative affective processing. Using a neuroimaging paradigm comprising two separate sessions recorded before and after 10 minutes of rest, we tested emotional reactivity upon reexposure to previously regulated negative emotional stimuli. We also tested whether neural activity within the control network during the initial presentation of the stimuli predicts subsequent affective processing. Thereby, we built on previous work of our group (Seidel et al.^48^), which reported on the first session of the experiment. That study contrasted the passive viewing of neutral and negative (disorder-irrelevant) pictures with the active down-regulation of emotional reactions by means of the reappraisal strategy ‘detachment’ (for details, see “Task and procedure” under “Methods” section, and supplementary information (SI) 1.2 and SI 1.4). Emotion regulation contrasts yielded an equally strong down-regulation of amygdala activation and self-reported arousal for healthy controls (HC) and young female AN patients. During passive viewing, however, AN patients showed significantly increased activation in the negative>neutral contrast in the left and right dlPFC as well as the right amygdala. In the current study, we extracted parameter values from these dlPFC clusters to predict neural activity linked to affective processing in the second session of the experiment (for details, see “Prediction analyses” in “Methods” section). In addition to ‘costs’ at the level of brain activation, we focused on the real-life implications of increased self-control. Using EMA, we tested for associations between the aforementioned neuroimaging markers and negative affect and tension in the two weeks following the experiment. We expected that increased neural activity within the control network, as frequently observed in AN including in our previous study^48^, would predict increased subsequent reactivity in brain regions pivotal to the processing of negative emotional stimuli, as well as subsequently increased negative affect and tension.

## Methods

### Participants

fMRI data were collected from 36 young female patients diagnosed with AN and a total of 45 female HC within the same general age range (mean: 16.6, range: 12–29 years). To optimize comparisons between AN and HC, we implemented a pairwise age-matching algorithm (for details, see SI 1.1) that yielded a final sample of *n* = 36 AN and *n* = 36 age-matched HC with a mean difference of 0.3 years (maximum: 0.8 years) within case-control pairs. Inclusion criteria for AN included a body mass index (BMI) below 17.5 kg/m^2^ (if younger than 15.5 years: below the 10th age percentile) and no recent weight gain. As only three AN participants (8.33%) were of binge-purge subtype, we did not conduct any subtype-specific analyses. HC had to have a body weight of >18.5 kg/m^2^ and <28 kg/m^2^ (or > 10th and <94th age percentile, if younger than 18 years), regular menstrual periods, and no history of psychiatric disorder. For all participants, eating-related psychopathology was assessed according to DSM-IV using the Structured Interview for Anorexic and Bulimic Disorders (SIAB-EX)^49^. Several other exclusion criteria were applied to both groups; most notably, bulimia nervosa or binge eating pathology, psychotropic medication, and neurological or medical conditions that may influence eating behavior or body weight (see SI 1.1). To supplement the information obtained through the SIAB-EX, we assessed eating disorder (ED) specific psychopathology (EDI-2^50^), depressive symptoms (BDI-II^51^), and anxiety levels (STAI^52^). BMI and BMI standard deviation scores corrected for age and gender (BMI-SDS^53^) are reported (see Table 1). An a priori power analysis was conducted using G*power^54^. In our previously published study we found group differences in the bilateral dlPFC reactivity during viewing of negative pictures between AN and HC which correspond to an effect size of *d* = 0.98 and 0.90^48^. Since we expected the effect size during reexposure to be smaller, we aimed to detect effect of at least *d* = 0.60, which corresponds to a sample size of *n* = 36 per group given an alpha-error probability of 5% and a power of 80%. The experimental procedure was approved by the ethics committee of the Technische Universität Dresden, and all participants (or their legal guardians) gave written informed consent.

**Table 1:** Basic demographic and clinical variables.

	HC (*n* = 36)	AN (*n* = 36)
Age (years)	16.65 (3.92)	16.52 (3.88)
BMI (kg/m^2^)	20.31 (2.28)	14.42 (1.23)***
BMI-SDS	−0.14 (0.55)	−3.35 (1.08)***
EDI-2	141.37 (24.88)	214.22 (46.21)***
BDI-II	5.41 (4.25)	22.25 (10.67)***
STAI-state	43.46 (5.84)	56.54 (8.81)***
STAI-trait	44.29 (6.81)	55.71 (9.30)***

*BMI* Body-mass-index, *BMI-SDS* BMI standard deviation score, *EDI-2* Eating Disorder Inventory, *BDI-II* Beck Depression Inventory, *STAI* State-Trait-Anxiety Inventory. Levels of significance are reported for independent sample *t*-tests between HC (healthy controls) and AN (Anorexia Nervosa patients); ***significant at *p* < 0.001.

### Task and procedure

Our analyses included data from three different experimental phases. In phase (1), fMRI during an emotion regulation task (fMRI-ER) was acquired. The resulting data were published in Seidel et al.^48^ and served as predictor variables in the current analyses (for details, see below and SI 1.2, SI 1.4 and SI 2.1). In phase (2), a reexposure fMRI task (fMRI-RX) required participants to passively watch a subset of the pictures previously presented in (1) fMRI-ER, as in Walter et al.^20^. The two fMRI sessions (phase (1) and (2)) were separated by a 10 minutes break in which structural scans were acquired. Phase (3) covered the 14 days that followed the MRI scan and encompassed EMA of tension and negative affect. Predictor variables from phase (1) fMRI-ER were available for 32 age-matched pairs. The incomplete sample overlap between phase (2) fMRI-RX (our sample) and phase (1) fMRI-ER (Seidel et al.^48^) was caused by partial data loss due to technical problems (3 HC) and the resulting need to run a separate age-matching algorithm (exchange of one HC). Data of the subsequent phase (3) EMA were available for all participants except three HC.

#### fMRI

Details on phase (1) fMRI-ER were published in Seidel et al.^48^. In short, the ER task contrasted the passive viewing of neutral and negative pictures (ER_neutral_watch_ and ER_negative_watch_) with the active down-regulation of emotional reactions to negative pictures (ER_negative_distance_). Participants were instructed to apply the reappraisal strategy ‘detachment’, which required them to distance themselves from any feelings elicited by the stimuli (for details, see SI 1.2). Pictures were selected from the International Affective Picture System (IAPS^55^) and the emotional pictures set^56^ and did not include any images related to food or body weight (see SI 1.2). Stimulus presentation was pseudorandomized for each participant so that conditions did not occur more than twice in a row. During phase (2) fMRI-RX, a subset of the pictures from (1) fMRI-ER was shown for 1 s each. Out of the 20 pictures of each fMRI-ER condition, 12 pictures were randomly chosen for each participant, resulting in a total of 36 fMRI-RX trials. An arousal rating was acquired after the presentation of each picture by means of a visual analog scale. The rating lasted for 3 s. Between trials, a fixation cross was presented for a jittered time interval (exponential distribution; mean = 3.5 s, range = 3–5 s). fMRI-RX took 7.5 min to complete. Analyses of fMRI-RX comprised the conditions RX_neutral (reexposure to pictures from ER_neutral_watch_) and RX_negative (reexposure to pictures from ER_negative_watch_ and ER_negative_distance_). During phase (2) fMRI-RX, the two groups of negative stimuli were presented with the same instruction to passively view the images and therefore subsumed into one condition. Supplemental analyses tested for sustained effects of ER (RX_negative_distance_ vs. RX_negative_watch_); that is, effects of the phase (1) fMRI-ER instruction that might carry over to passive reexposure in phase (2) fMRI-RX. Those analyses were not the main focus of this study, did not yield any significant results and are thus reported in SI 2.4 only.

In accordance with guidelines for brain imaging investigations in eating disorder populations^57^, we acquired all MRI data between 8 and 9 AM following an overnight fast to control for acute nutritional intake and diurnal hormone rhythms. AN participants were tested within 96 hours after admission to a behaviorally oriented nutritional rehabilitation program. Imaging was carried out with a 3 T whole-body MRI scanner (TRIO; Siemens, Erlangen, Germany) equipped with a standard 12 channel head coil. The functional images were recorded with a gradient-echo T2*-weighted EPI sequence with the following parameters: number of volumes = 210, number of slices = 42, repetition time = 2410 ms. Structural brain scans were acquired with a T1-weighted rapid acquisition gradient echo (MP-RAGE) sequence (number of slices = 176, repetition time = 1900 ms, slice thickness of 1 mm). For more details, see SI 1.2.

#### EMA

A comprehensive description of experimental phase (3), EMA data collection, can be found in two previous studies of our group^58,59^ and in SI 1.3. EMA took place during a period of 14 days following the MRI scan. Participants were prompted to answer an app-based questionnaire via smartphone at six semi-random times per day. At each prompt, current tension and negative affect were assessed with two bipolar items each. The items are part of an adapted version of the Multidimensional Mood Questionnaire (MDMQ^60^) recommended for the use in EMA research^61^. Answers were given on continuous rating scales with opposite anchors (e.g., “agitated-calm”). Additionally, participants reported their main activity since the last prompt and the company of other people for the purpose of contextual control.

### Data analysis

#### Reexposure task

##### Arousal ratings

Analyses of arousal data and clinical variables were performed in SPSS (Version 25.0, IBM Corp., Armonk, NY, USA). Missing arousal ratings were excluded from the analysis (<0.6% in all conditions). A univariate mixed-design ANOVA was conducted with all valid ratings, including the between-subject factor group (AN, HC) and the within-subjects factor condition (RX_neutral, RX_negative). All ANOVA reports in this study encompassed Greenhouse-Geisser corrected F statistics^62^ and partial eta squared (η^2^) as measures of effect size. Levene’s test was used to ensure homogeneity of group variances.

##### MRI Preprocessing

The functional data were preprocessed in SPM 8 (Statistical Parametric Mapping software, https://www.fil.ion.ucl.ac.uk/spm/) within the Nipype framework^63^. Quality of the fMRI data was assessed using visual inspection and artefact detection tools (ART, www.nitrc.org/projects/artifact_detect/). Functional images were checked for motion outliers (>2 mm in any direction) and intensity outliers (>3 SD above the mean of the time series). The number of outliers was below 6.2% of all frames for all participants (mean: 1.85%, SD: 1.53%) and did not differ between AN and HC (intensity-outliers: *t* = 0.219, *p* = 0.827; motion-outliers: *t* = 0.692, *p* = 0.492). Realign4D was used to correct the functional images for head motion and staggered slice acquisition. The algorithm allows for an improved reduction of spatio-temporal distortion by simultaneously accounting for the combination of slice-timing and realignment^64^. The functional images were first co-registered to the participant’s structural T1-image. Subsequently, they were normalized to MNI space by means of a group template created in DARTEL^65^. The DARTEL template included the structural images of both AN and HC to provide a reference unbiased by potential group differences. Finally, an isotropic Gaussian smoothing kernel with 8-mm full-width at half-maximum was applied.

##### MRI Analysis

Modeling and analysis of fMRI data from the ER phase are described in Seidel et al.^48^ and SI 1.4. For individual participants’ RX data, a general linear model was fit to each voxel’s hemodynamic response in the two experimental conditions: (i) RX_neutral (ii) RX_negative. We modeled the presentation of the pictures as a boxcar function with a duration of 1 second and the subsequent rating as a boxcar function of 3 s. Additionally, the six realignment parameters and one regressor for each motion or intensity outlier volume were included as nuisance regressors of no interest. On the level of group analysis, a linear mixed model was estimated using SPM 12. This model included a binary within-subject variable (condition: neutral, negative) and a binary between-subject variable (group: AN, HC). Contrasts were set up to explore the main effect of group and condition as well as their interaction. We focused on five previously defined brain regions of interest (ROI) that are pivotal in the processing and regulation of (negative) emotional stimuli^66–68^: amygdala, insula, ventromedial prefrontal cortex (vmPFC), dorsolateral prefrontal cortex (dlPFC), and anterior cingulate cortex (ACC). Information on mask creation is provided in SI 1.4. Additional whole brain analyses are provided in Supplementary Table 1. Age was included as a covariate in all analyses. Correction for multiple comparisons was accomplished via small volume correction performed in the updated version of 3DClustSim, released June 2017—for details, see SI 1.4. For use in the prediction analyses, mean parameter estimates (beta values) were extracted with MarsBar toolbox for SPM^69^.

#### Prediction analyses

##### Prediction of RX-MRI data by ER-MRI data

In order to investigate whether data of phase (2) fMRI-RX could be predicted by data from the earlier phase (1) fMRI-ER, we conducted a series of regression analyses within the PROCESS toolbox (version 3.0^70^) in SPSS (Version 25.0, IBM Corp., Armonk, NY, USA). Predictor variables were mean parameter estimates derived from activation in the dlPFC clusters in which we observed a significant group effect (AN > HC) in phase (1) fMRI-ER in response to negative pictures (ER_negative_watch_ > ER_neutral_watch_, see Seidel et al.^48^). The clusters were labeled right dorsal dlPFC, left ventral dlPFC and left dorsal dlPFC (**х̅**ER_dlPFC_Ldorsal_, **х̅**ER_dlPFC_Lventral_, **х̅**ER_dlPFC_Rdorsal)_. To test the regional specificity of the results in a control analysis, the activation of the right amygdala (х̅ER_amygdala_R_) identified by the same contrast in our previous study (Seidel et al.^48^) also served as a predictor. For details about the predictor variables’ definition, see SI 1.4 (analyses) and SI 2.1 (results). In addition to activation in these predictor regions, diagnostic group and the interaction between diagnostic group and regional activation served as predictors in each analysis. Outcome variables were parameter estimates extracted from clusters with significant group effects identified by phase (2) fMRI-RX contrasts. Since the fMRI-RX group effects were all condition-unspecific, fMRI outcome as well as fMRI predictor variables were both computed as mean extracted parameter estimates (across all conditions). For all analyses, the assumption of homoscedasticity was confirmed by the Breusch-Pagan test.

##### Prediction of EMA data

Since the EMA assessment yielded a nested data structure, we conducted hierarchical linear modeling (HLM) to investigate whether data of phase (2) fMRI-RX predicted phase (3) EMA measurements of negative affect and tension 14 days following the scan. Affect and tension served as outcome variables in separate HLM models. The models took into account that the EMA data set was organized in three different levels. Single observations (Level A) were nested within days (Level B) which were nested within participants (Level C). All models allowed for random intercepts and included the following control variables: day of study (1–14) on Level B, time (time of the day as a continuous variable from 1 to 6) and the dummy-coded variables of current company and primary activity (chosen from six categories) on Level A. On Level C, we included diagnostic group (coded −1 for HC and 1 for AN), individual parameter estimates of the clusters with a phase (2) fMRI-RX group effect, and their interaction.

## Results

### Demographic and clinical variables

As shown in Table 1, AN and HC did not differ with regard to age. As expected, AN had a lower BMI, higher eating disorder (EDI-2), depressive (BDI-II), and anxiety symptoms (STAI-state and STAI-trait) when compared with HC.

### Reexposure task

As expected in this task design, mean arousal ratings during phase (2) fMRI-RX were significantly lower for neutral compared to negative stimuli (F(1, 70) = 358.18, *p* < 0.001, *η*^2^ = 0.84). There was no significant effect of group (F(1,70)=1.78, *p* = 0.19, *η*^2^ = 0.03) or interaction between group and condition (F(1, 70) = 0.19, *p* = 0.66, *η*^2^ = 0.003); for means and standard deviations, see Supplementary Table 2. Results of the ROI analyses of the phase (2) fMRI-RX data are displayed in Table 2. Confirming that the RX task worked as expected, viewing negative compared to neutral pictures elicited increased activation in the right amygdala and left insula in both groups. Compared to HC, AN showed increased activation of the right and left amygdala across all conditions. There was no significant interaction between the factors condition and group. In order to ensure that group differences in amygdala activation resulted from core pathology of AN rather than comorbid anxiety symptoms, a separate control analysis included trait anxiety scores (STAI-trait) as a covariate. Group differences remained significant with comparable effect sizes (left amygdala: F(1, 61)=21.33, *p* < 0.001, *η*^2^ = 0.26; right amygdala: F(1, 61) = 12.77, *p* = 0.001, *η*^2^ = 0.17). Furthermore, levels of STAI-trait did not correlate significantly with left or right amygdala activation in any of the two groups, which speaks against a confounding influence of trait anxiety symptoms.

**Table 2:** Results of ROI analyses for phase (2) RX-fMRI data.

Contrast	ROI	H	XYZ	K	Zmax
Main effects of group					
AN > HC	Amygdala	R	26 −4 −16	100	5.25
		L	−24 −6 −14	57	3.93
HC > AN	–	–	–	–	–
Main effects of condition					
Negative > Neutral	Amygdala	R	24 2 −24	9	3.54
	Insula	L	−36 24 8	28	4.26
Neutral > Negative	Insula	R	8 12 −12	72	4.87
	dlPFC	R	28 40 42	74	3.50

*H* hemisphere, *XYZ* MNI coordinates, *K* cluster size (number of voxels), *Zmax* peak z-value, *HC* healthy controls, *AN* Anorexia nervosa patients. All clusters survived whole-brain FWE correction at *p* < 0.05, with a voxel-wise threshold of *p* < 0.001 and ROI-specific cluster extent thresholds (see SI 1.4).

### Prediction analyses

#### Reexposure task

**Fig. 1:**
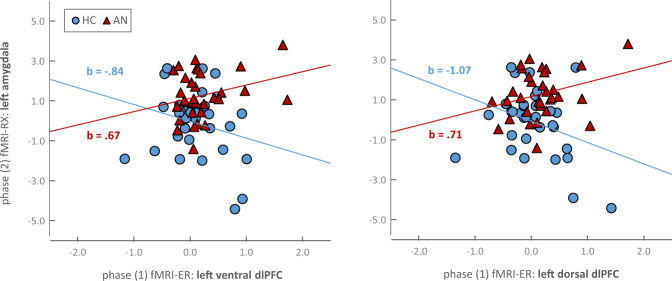
Prediction of phase (2) fMRI-RX left amygdala activation (х̅RX_amygdala_L_) by phase (1) fRMI-ER left ventral dlPFC and left dorsal dlPFC activation (х̅ER_dlPFC_Lventral_ and х̅ER_dlPFC_Ldorsal_), moderated by group. The linear b coefficients were significantly increased in AN compared to HC (left ventral dlPFC: *t*(60) = −2.02; *p* = 0.048; left dorsal dlPFC: *t*(60) = −2.54, *p* = 0.014).

As can be seen in Table 3, phase (1) left dorsal dlPFC activation (**х̅**ER_dlPFC_Ldorsal_) as well as phase (1) left ventral dlPFC activation (**х̅**ER_dlPFC_Lventral_) differentially predicted phase (2) left amygdala activation (**х̅**RX_amygdala_L_) as a function of diagnostic group: in AN, higher levels of phase (1) left dorsal dlPFC and left ventral dlPFC activation (**х̅**ER_dlPFC_Ldorsal_ and **х̅**ER_dlPFC_Lventral_) predicted higher levels of phase (2) left amygdala activation (**х̅**RX_amygdala_L_). In HC in contrast, higher levels of phase (1) left dorsal dlPFC and left ventral dlPFC activation (**х̅**ER_dlPFC_Ldorsal_ and **х̅**ER_dlPFC_Lventral_) predicted lower levels of phase (2) left amygdala activation (**х̅**RX_amygdala_L_). For illustration of the moderation effects, see Fig. 1. Phase (1) activation of the right dorsal dlPFC (**х̅**ER_dlPFC_Rdorsal_) did not have a predictive value for phase (2) amygdala activation. Suggestive of an effect specific to brain regions associated with cognitive control, phase (1) activation of the right amygdala (**х̅**ER_amygdala_R_) did not have a predictive value for phase (2) amygdala activation (for details, see Table 3).

**Table 3:** Results of regression analyses: Prediction of phase (2) fMRI-RX amygdala activation by phase (1) fMRI-ER dlPFC activation and, as a control analysis, by phase (1) fMRI-ER amygdala activation, moderated by group.

		Phase (2) fMRI-RX: dependent variable			
		х̅RX_amygdala_L_		х̅RX_amygdala_R_	
		*beta* ± *SEM*	*p*	*beta* ± *SEM*	*p*
Phase (1) **fMRI-ER: predictor**	**Left ventral dlPFC**				
	Constant	−0.02 ± 0.26	0.93	−0.14 ± 0.34	0.68
	х̅ER_dlPFC_Lventral_	−0.84 ± 0.53	0.12	−0.49 ± 0.48	0.48
	Group	1.15 ± 0.38	<0.01**	1.77 ± 0.50	<0.001***
	Group*х̅ER_dlPFC_Lventral_	1.51 ± 0.75	<0.05*	0.30 ± 0.99	0.77
	**Left dorsal dlPFC**				
	Constant	−0.07 ± 0.25	0.77	−0.16 ± 0.33	0.64
	х̅ER_dlPFC_Ldorsal_	−1.07 ± 0.49	<0.05*	−0.92 ± 0.65	0.16
	Group	1.24 ± 0.36	<0.01**	1.65 ± 0.48	<0.01**
	Group*х̅ER_dlPFC_Ldorsal_	1.78 ± 0.71	<0.05*	1.51 ± 0.95	0.12
	**Right dorsal dlPFC**				
	Constant	−0.06 ± 0.26	0.82	−0.21 ± 0.35	0.54
	х̅ER_dlPFC_Rdorsal_	−0.35 ± 0.38	0.37	−0.14 ± 0.50	0.78
	Group	1.31 ± 0.37	<0.001***	1.80 ± 0.49	<0.001***
	Group *х̅ER_dlPFC_Rdorsal_	0.52 ± 0.50	0.30	−0.16 ± 0.65	0.80
	**Right amygdala**				
	Constant	−0.09 ± 0.26	0.74	−0.15 ± 0.34	0.65
	х̅ER_amygdala_R_	−0.49 ± 0.96	0.61	−0.73 ± 1.24	0.57
	Group	1.39 ± 0.37	<0.001*	1.72 ± 0.48	<0.001*
	Group *х̅ER_amygdala_R_	−0.06 ± 1.20	0.96	1.09 ± 1.56	0.49

Note: All fMRI variables are mean parameter estimates (across conditions). *SEM* standard error of the mean. *significant at α ≤ 0.05; **significant at α ≤ 0.01, ***significant at α ≤ 0.001.

#### EMA data

HLM results are displayed in Table 4 and Supplementary Table 3 (full model). For use within the HLM, individual fMRI-RX parameter estimates of the significant left and right amygdala clusters were averaged to serve as a single predictor variable at the level of participants (Level C). The momentary level of tension in the two weeks after the fMRI experiment was significantly predicted by group and phase (2) fMRI-RX amygdala reactivity. HC reported generally less tension than AN. Participants with higher fMRI-RX amygdala reactivity reported increased tension compared to those with lower amygdala reactivity. The interaction term was not significant, however, indicating that the size of the amygdala effect did not vary by group. Negative affect was significantly predicted by group, but not phase (2) fMRI-RX amygdala activity, with HC reporting less negative affect than AN. These results were validated by control analyses accounting for potentially confounding effects of compliance with the EMA protocol (see Supplementary Table 3).

**Table 4:** Non-standardized β-values of the EMA HLM analyses.

Predictor (Level C)	Phase (3) EMA outcome variable	
	Tension	Negative Affect
Group	−13.61*	−33.06**
Phase (2) fMRI-RX amygdala	−7.67**	−2.71
Phase (2) fMRI-RX amygdala * group	−2.54	1.66

Notes: Phase (3) tension and negative affect predicted by group, phase (2) fMRI-RX amygdala activation, and their interaction (Level C). Phase (2) fMRI-RX amygdala activation was averaged over the hemispheres. Predictors of Level B (day of study) and Level A (time of day, company, dummy-coded variables for activity) are not displayed; full model can be seen in SI Table 3. Negative affect and tension as measured by EMA questionnaire; lower values indicate more tension and more negative affect. Group was coded −1 = HC (healthy controls), 1 = AN(Anorexia nervosa patients). *N* = 69. *significant at α ≤ 0.05; **significant at α ≤ 0.01.

## Discussion

The aim of the current study was to explore whether over-control in AN, as gauged by increased dlPFC activation, may be associated with costs in the domain of affective processing. Indeed, higher dlPFC activation during emotion processing predicted increased amygdala reactivity at reexposure after a 10 min break in AN, but not HC. Increased amygdala reactivity itself was related to increased self-reported momentary tension in everyday life during two weeks following the scan. As discussed below, these data suggest that over-control in AN may result in negative affective states on a short (minutes) as well as a longer time scale (days), a mechanism that may significantly contribute to the persistence of AN symptomatology.

In detail, fMRI data revealed increased reactivity of the bilateral amygdala in AN compared to HC that was present during passive reexposure to previously watched negative and neutral pictures. Increased amygdala activity is a commonly observed neural signature of emotional arousal^71^. In AN patients, heightened amygdala reactivity has previously been reported in response to disorder-related^72–74^ and disorder-unrelated emotional stimuli^48^. As the observed increase in amygdala activation was not specific to one of the conditions (i.e., present when viewing negative and neutral pictures), we assume that it may reflect a general susceptibility for negative affective states in AN. Quantitative resting state data also support an increased amygdala blood flow in AN^75^.

Capitalizing on the multi-session design of our fMRI paradigm, we showed that amygdala reactivity during reexposure could be predicted by left ventral dlPFC and left dorsal dlPFC activation during the preceding, initial exposure to the stimuli. Importantly, this effect was specific to the dlPFC: a control analysis demonstrated that preceding amygdala activation could not explain amygdala activation at reexposure (see Table 3). The direction of this relationship depended upon group. In HC, higher dlPFC activation predicted lower amygdala activation during reexposure; a pattern that is also known from multi-session fMRI recordings of successful ER in HC^20^. In AN, however, higher dlPFC activation predicted higher subsequent amygdala activation. The dlPFC is considered a crucial brain region of the top-down control network which helps to initiate and adjust control of behavior as well as internal states^13,14^. In our data, group-specific dlPFC effects occurred under conditions that did not explicitly require heightened self-control or cognitive control^48^. Therefore, we suggest that the heightened amygdala reactivity in AN may be a cost of implicit control mechanisms that are activated automatically, or a cost of a heightened baseline level of sustained cognitive control^42,76^. Our results extend previous findings of increased fronto-parietal control network activation in AN employing disorder-related and unrelated stimuli^37–41,77,78^. They are in line with the proposition that the increased control network activity in AN is partly independent of task and stimulus type, as also reflected in resting state fMRI data^42–44^. However, it is also important to consider the heterogeneous nature of previous dlPFC findings from voluntary ER experiments in AN. While Seidel et al.^48^ reported dlPFC hyper-activation in AN, Steward et al.^45^ described dlPFC hypo-activation. The apparently discrepant findings of these studies are difficult to compare, however, because of noteworthy differences including age, task design and analysis (event- vs. block-related), satiation, the timepoint of scanning in relation to treatment begin; all of which are generally important factors to consider when interpreting neuroimaging results in ED studies^57^. Ironically, our fMRI data suggest that ‘preventive over-control’ may backfire, making states of increased arousal more likely to occur. This is supported by our EMA measurements which, like previous findings^79^, indicate higher self-reported momentary tension in AN. We found that EMA ratings could be predicted by amygdala reactivity during reexposure. Controlling for a large number of context variables (such as current activity and the company of others), these findings establish a link between neural activity in a controlled laboratory task and disorder-relevant affective states in real-life situations over the fairly long time course of two weeks.

Evidence that over-control may come at a cost such as increased tension in everyday life is in line with EMA data showing that highly restrictive eating coincides with higher tension, anxiety and negative affect measured throughout the day^80^. These results offer an insightful perspective on the clinical presentation of AN. Several theoretical models of AN etiology include heightened tension and highly controlled behavior as core features^25–28^. However, these models state that over-controlled behavior (e.g., dietary restriction) *reduces* tension^28^, presumably serving as an alternative, maladaptive strategy of ER^27^. In accordance with Haynos et al.^80^, our results question the effectiveness of over-control (including emotional avoidance) as an ER strategy and suggest that while over-controlled behavior may be experienced as a helpful way to reduce tension in the short term, it may in fact *increase* tension in the long run. This possibility is supported by data on the downregulation of positive emotions, which has been shown to predict higher body-related rumination and increased negative affect in AN^47^. In other studies, higher daily levels of negative affect in AN predicted increased rumination^58^ and increased dietary restriction on the following day^81^. In light of these findings, it seems plausible that negative affective states and a tendency towards over-controlled behavior form a self-perpetuating circle that impedes the reduction of dysfunctional behavior in AN and prevents long-term improvement of mood. From a methodological point of view, our findings emphasize the importance of precisely defining time scales (e.g., short-term versus long-term regulation) when investigating ER in eating disorders. As also pointed out by Engel et al.^81^, temporal parameters of analytic approaches could account for some of the previous inconsistencies in ER studies on AN. From a conceptual point of view, our findings also highlight complexities in the definition of self-control^82,83^. While self-control is often viewed as a limited resource that costs effort^5^, others describe it as a dynamic process of resolving goal conflict, e.g., between have to and want to goals or short- and long- term goals^83–85^. From this perspective, restrictive eating in AN can be viewed as a consequence of the conflict between hedonic eating and weight control being resolved by heightened accessibility of long-term (weight-related) goals—an implicit process which might enable AN patients to control themselves even in very tempting situations^86,87^. Thus, self-control may be ‘second nature’ for many individuals with AN. Similarly, constant down-regulation of emotions may emerge from the goal conflict between acting out and down-regulating emotions being implicitly resolved by self-restraint. In the context of goal conflict theories, these behavioral tendencies in AN may be seen as maladaptive because they fail to establish a balance between different types of goals. This predominance of weight/emotion control goals has severe long-term physical and emotional consequences^31^.

Similar costs of over-control have also been observed in healthy individuals who showed increased amygdala reactivity in response to emotional stimuli after performing a demanding attention control task^19^. Amygdala reactivity also seems to be amplified in healthy participants who habitually use emotion suppression^20,88^. Furthermore, control strategies such as thought suppression have been shown to aggravate the desire to overeat in normal-weight and overweight restrained eaters^89,90^ and worsen clinical symptoms in patients with bulimia nervosa (BN)^91,92^. In the latter two groups, phases of dietary restriction alternate with loss of control and food binging, an effect that seems to be reinforced by negative emotions^93,94^. Although only 8.33% of the AN patients in the current study were of the binge-purge subtype, it is possible that the different aforementioned expressions of costs of over-control are grounded in similar interactions between amygdala and dlPFC as observed in our AN sample. This possibility should be addressed by future studies.

The results of our study have to be considered in the light of the following limitations. Amygdala reactivity predicted EMA measures of momentary tension, but not negative affect. Possible explanations are a stronger involvement of the amygdala in the processing of tension and arousal compared to affective valence^95,96^, and potentially biasing effects of social desirability or alexithymia^97^ on the valence ratings of AN patients. Another variable that might have had undue influence on our results is the fairly broad age range of the sample (12-29 years). Despite our strict, pair-wise age-matching and the inclusion of age as a covariate in all analyses, an influence of (neuro-) developmental factors cannot be excluded. During adolescence, the dlPFC undergoes a critical phase of cortical maturation and thinning^98^, which has been associated with changes in the use of cognitive control and ER^99,100^. These processes, as well as detrimental effects of chronification with longer durations of illness, might lead to different results in an older AN sample. Additionally, it remains unclear to what extent altered amygdala activation is caused by the state of undernutrition in AN patients^24,101^. Since it has been suggested that abnormalities in amygdala reactivity might persists beyond recovery of AN^102,103^, similar studies in weight-recovered, former patients might help to disentangle state and trait factors.

Our data extend the hypothesis of sustained and elevated self-control in AN^27,28^ by providing insight into the costs of over-control. They suggest that heightened activity of control-related brain regions is associated with increased negative affective processing, supporting the notion that AN may be characterized by an altered balance between ventral limbic and dorsal executive networks^28,104^. Over-controlled behavior and negative affective states may reinforce each other^81^, a mechanism that could contribute to the maintenance of AN symptoms. From a clinical perspective, these results lend support to therapeutic approaches that enable AN patients to better allocate the use of self-control. Helpful techniques may for instance be derived from the “radically open-dialectical behavior therapy”, which specifically targets overly controlled behavior and has been successfully tested in the treatment of AN^105,106^. Moreover, therapeutic approaches that are based on mindfulness and emotional acceptance may tip the balance between hedonistic, acceptance-based processing and depleting, control-based strategies in a favorable manner^107^. In the treatment of severe enduring AN, repetitive transcranial magnetic stimulation (rTMS) to the left dlPFC has been associated with improvements in ED symptoms, including a reduction of self-controlled food choice and affective symptoms^108,109^, which were maintained at an 18 month follow-up^110^. Taken together, our results provide evidence in support of these therapeutic approaches and may stimulate further research into interventions that modulate the balance between affective and executive control networks.

## Supplementary information

Supplementary Information
