# Supplementary material for: The costs of over-control in anorexia nervosa: evidence from fMRI and ecological momentary assessment
Source: Transl Psychiatry. 2021 May 21;11:304. doi: 10.1038/s41398-021-01405-8 (PMC8138008; doi:10.1038/s41398-021-01405-8)
Supplement: Supplementary file 1 — Supplementary Information [file 41398_2021_1405_MOESM1_ESM.docx]

The costs of over-control in anorexia nervosa: evidence from fMRI and ecological momentary assessment

Supplementary Information

[1. Methods 2](#_Toc69974189)

[1.1. Participants 2](#_Toc69974190)

[1.2. MRI task and procedure 2](#_Toc69974191)

[1.3. Ecological Momentary Assessment (EMA) 3](#_Toc69974192)

[1.4. Data analysis 4](#_Toc69974193)

[2. Results 5](#_Toc69974194)

[2.1. fMRI-ER task 5](#_Toc69974195)

[2.2. fMRI-RX task: exploratory whole-brain analysis 6](#_Toc69974196)

[2.3. fMRI-RX task: arousal ratings 7](#_Toc69974197)

[2.4. fMRI-RX task: sustained effects of emotion regulation 7](#_Toc69974198)

[2.5. Prediction of EMA data 7](#_Toc69974199)

[References 8](#_Toc69974200)

## **Methods**

## **Participants**

Pair-wise case-controlled age-matching was based on the Munkres algorithm^1^, resulting in a maximum difference of 0.8 years between one pair of participants (mean: 0.3 years). In line with this, AN (mean=16.52, SD=3.89) and HC (mean=16.55, SD=3.92) did not significantly differ with regard to age (t=0.14, p=.88). AN participants were inpatients at a child and adolescent psychiatry or psychosomatic medicine department. They underwent the assessment within 96 hours after admission to a behaviorally oriented nutritional rehabilitation program. HC were recruited through public advertisement in schools and universities. HC had to have normal body weight (BMI >18.5kg/m^2^ and <28kg/m^2^, or >10th and <94th age percentile, if younger than 18 years) and a regular menstrual period. The recruitment process and all psychological assessments were conducted by clinically experienced research assistants under the supervision of a senior child and adolescent psychiatrist. HC were excluded if they reported any history of psychiatric illness. In AN, exclusion criteria comprised any history of bulimia nervosa or binge eating, substance abuse, schizophrenia, psychosis or bipolar disorder. Five AN were diagnosed with comorbid psychiatric disorders (including depressive episodes (n=2), obsessive compulsive disorder (n=1), social phobia (n=1), and undifferentiated somatoform disorder (n=1). Comorbid diagnoses in AN were made by an expert clinician following a careful, interdisciplinary diagnostic procedure during the inpatient treatment. Psychiatric diagnoses in HC were excluded by means of a semi-structured interview. This interview also included questions on further exclusion criteria in both experimental groups, namely psychotropic medication (up to four weeks prior to the study) and inflammatory, metabolic, neurological, or other chronic disorders with potential influence on appetite, eating behavior, or body weight (such as diabetes, thyroid disease, organic brain syndrome). Furthermore, participants of both groups were excluded if they were currently pregnant, breast-feeding, or had an intelligence quotient <85. For intelligence diagnostics, we applied a short version of the German adaptations of the Wechsler Adult Intelligence Scale^2^ or the Wechsler Intelligence Scale for Children^3^. Participants were paid for their participation.

## MRI task and procedure

**Stimulus material.** The selection of pictures was based on the International Affective Picture System (IAPS^4^) and the emotional pictures set^5^ and did not include any pictures related to food, female bodies or physical exercise carried out ‘to burn calories’. A pilot study on 15 AN showed that valence and arousal ratings were highly similar to the two studies providing the stimulus material^6^.

**Instructions and set-up of the ER task.** In the passive viewing conditions of the phase (1) emotion regulation (ER) task (ER_neutral_watch_, ER_negative_watch,_ and ER_positive_watch_), participants were asked to view pictures for the whole time, without distracting themselves or modulating upcoming feelings. Note that conditions with positive stimuli were not analyzed in the current study. For results of the ER task with positive images, please refer to Seidel et al.^7^ In the ER conditions (ER_negative_distance_ and ER_positive_distance_), participants were instructed to distance themselves from any feeling elicited, for example by taking the position of a neutral, non-involved observer. The participants were encouraged to use their imagination (e.g. visualizing the picture to become smaller or being situated behind a glass wall), but to keep looking at the picture for the whole time. A training session outside the scanner ensured a sufficient understanding of the instruction; the 17 pictures from the training session were not used in the scanner. In the ER experiments, pictures were shown for a total of 7.5 seconds, with the instruction to “view” or “distance” overlaying the stimulus for 1.5 seconds at the beginning of the presentation. A momentary arousal rating was acquired after the presentation of each picture, applying a visual analogue scale ranging from “not aroused at all” to “very aroused”. The rating lasted for 3 seconds. 20 trials were presented for each condition, resulting in a total of 100 trials. The phase (1) fMRI-ER task took 22.5 minutes to complete.

**Data Acquisition**. Task presentation and recording of the behavioral responses were carried out via Presentation (Neurobehavioral Systems, Inc., Albany, CA). Stimuli were presented onto a mirror-based back-projection screen attached to the head coil of the scanner. The functional images were recorded with a gradient-echo T2*-weighted EPI sequence (TR=2410 ms; TE=25 ms; flip angle=80°). A total of 210 volumes were obtained (42 transversal slices orientated 17° clockwise to the AC-PC line, 2 mm slice thickness, 1 mm gap, FOV=192×192 mm, in-plane resolution of 64×64 pixels=voxel size of 3×3×2 mm^3^). Structural brain scans were acquired with a T1-weighted rapid acquisition gradient echo (MP-RAGE) sequence (TR=1900ms, TE=2.26ms, FOV=256×256 mm, 176 slices, 1×1×1 mm^3^ voxel size, flip angle=9°). The web-based electronic data capture tool REDCap^8^ was used to manage data collection.

## Ecological Momentary Assessment (EMA)

Data were either collected via a study smartphone (Samsung Galaxy Ace, 5831 i) or via the participant’s own smartphone (which was the case in n=1). The online platform Movisens XS (Karlsruhe, Germany) was used to design the questionnaire, manage data collection and provide an immediate data upload for constant monitoring of compliance. Current tension and negative affect were assessed with two questions each. The items were adapted from the multidimensional mood questionnaire (MDMQ^9^) and required a continuous rating on the following scales: “agitated-calm” and “relaxed-tense” (tension); and “content-discontent” and “well-unwell” (negative affect). Besides the MDMQ-scales for tension and negative affect, three more variables were assessed with each prompt: “energetic arousal” (the third scale of the MDMQ, which assesses “awakeness-tiredness”) and rumination about food and weight (two questions adapted from the SIAB-EX^10^). Additionally, several contextual variables were assessed. To control for possible situational influences on the data, participants reported whether they had mainly been alone vs. in company since the last prompt, whether they had been mainly at home vs. elsewhere, and what their main activity had been. For the latter, a choice of several different categories was possible: e.g. leisure, therapy, eating, sleep, other.

EMA sampling started the day after the fMRI scan and lasted for a period of 14 days. Beforehand, participants received detailed instructions on how to use the smartphone, the MovisensXS-app, and how to handle the questionnaire. EMA sampling was accomplished via the signal-contingent assessment method: Alarms occurred at six semi-random times a day within a period of 14 hours. The start time was individually adapted to suit different daily routines. Participants were instructed to answer each questionnaire as soon as it appeared, but allowed to postpone the alarm for a maximum of 30 minutes if they were unable to answer instantly. To minimize interference with inpatient treatment, prompts were anchored within six smaller intervals of 2x2 ½ hours (before midday) and 4x1 ½ hours (after midday) which were separated by 30 minute breaks. During the first days of participation, recordings were closely monitored in order to ensure compliance and understanding of the instructions. Financial compensation was provided after the end of the study and partly depended on individuals’ compliance rate.

## Data analysis

**Region of Interest (ROI) analyses.** The anatomical masks of amygdala and insula were created by merging the corresponding regions of the Automated Anatomic Labelling (AAL) atlas provided within the Wake Forest University (WFU) PickAtlas for SPM^11,12^. For the left and right dlPFC, two separate masks were created by merging the regions termed superior and middle frontal gyrus in the WFU PickAtlas, but parts posterior to y=24 (MNI space) were excluded as in our previous work^13,14^. The vmPFC mask was created by merging the left and right frontal medial orbital cortex label from the WFU PickAtlas. The mask of the ACC was created by intersecting a sphere (centered at x=-2, y=22, z=34, radius=32 mm) and a binarised probabilistic map (>0)^15^ from the Harvard-Oxford atlas^16^.

**Small volume correction via 3DClustSIM.** Correction for multiple comparisons was accomplished via small volume correction performed in the updated version of 3DClustSim, released June 2017 (<http://afni.nimh.nih.gov/pub/dist/doc/program_help/3dClustSim.html>). Based on Monte Carlo Simulations, this program estimates the cluster size at which the chance of false positives falls below a given alpha level (α=.05, two-sided) for a given voxelwise p-value level (p=.001). Separate simulations were run for each ROI. A cluster was considered significant if it exceeded the following minimum cluster sizes: amygdala k=2, insula k=23, VS k=4, ACC k=29, lDLPFC k=26, rDLPFC k=30, vmPFC=5.

**Sustained effects of ER during RX.** An additional set of analyses was run to test for sustained effects of emotion regulation; that is, effects of the phase (1) fMRI-ER instruction (ER_negative_watch_ vs. ER_negative_distance_) that might carry over to passive reexposure in phase (2) fMRI-RX (RX_negative_watch_ vs. RX_negative_distance_). The applied set-up was identical to the one described in the main manuscript (paragraph 2.4.1 to 2.4.2) with the following exceptions. For analyses of arousal ratings, a univariate mixed-design ANOVA was conducted with the between-subject factor group (AN, HC) and the within-subjects factor previous instruction (RX_negative_watch_, RX_negative_distance_). For analysis of the individual fMRI data, a general linear model was fit to each voxel’s hemodynamic response in the experimental conditions i) RX_negative_watch_ ii) RX_negative_distance_. The model for group level analysis included a binary within-subject variable (instruction: watch, distance) and a binary between-subject variable (group: AN, HC). Contrasts were set up to explore the main effect of group and instruction as well as their interaction.

**Analysis of fMRI-ER data.** Our current analyses include predictor variables derived from the fMRI-ER data. The analysis of the fMRI-ER data has been reported previously by Seidel et al. (pp. 3-4)^6^ and is described briefly below:

“Statistical analysis of the fMRI data involved fitting a general linear model (GLM) separately for each participant on a voxelwise basis to model the hemodynamic response to each of the five conditions (neutral, positive/negative watch, positive/negative distance). We modeled the picture presentation phase as boxcar function with a duration of 6 s and the subsequent rating as a stick-function (zero duration). Additional regressors included six motion parameters and one regressor for each motion or intensity outlier volume as nuisance regressors of no interest. All events were modeled using a canonical hemodynamic response function. Based on previous studies of negative emotion regulation (Ochsner et al., 2012; Walter et al., 2009) and our research question of possibly altered emotional reactions to aversive pictures in AN, we focused on the analysis of the negative pictures [for results of the regulation of positive images please refer to Seidel et al.^7^]. To confirm that the emotion induction worked as intended, we first examined specific activation patterns within the watch conditions by calculating the contrasts negative watch > neutral watch. To address the main research question of this study regarding potential group differences during the regulation condition, we examined the contrasts negative watch > negative distance and negative watch < negative distance. At the second level, we conducted independent two-sample t-tests to assess group differences between these individual contrasts within a priori anatomically defined regions of interest (ROIs): the bilateral amygdala and dlPFC, as defined by the AAL atlas (Maldjian et al., 2003; Tzourio-Mazoyer et al., 2002) and implemented in the WFU PickAtlas toolbox for SPM (Maldjian et al., 2003) using MarsBaR toolbox for SPM (Brett et al., 2002). To control for false positives, familywise error correction was performed using 3DClustSim (version from 3rd of July 2017; http://afni.nimh.nih.gov/pub/dist/doc/program_help/3dClustSim.html). Specifically, the program was used to run 10,000 Monte Carlo simulations to estimate the cluster size above which the false positive probability is below a given α-level (α=0.05) for a given voxel-wise p value, which was set at 0.001. At this voxel-wise threshold (two-sided), clusters with more than six voxels for the left and right amygdala and 34 voxels for the left and right dlPFC each corresponded to a combined threshold of p<0.05 [familywise error (FWE) corrected] in the respective ROI. The details of supplemental exploratory whole-brain analyses are provided in the supplementary material (Fig. S1).

To calculate associations between neural activation in the different conditions and psychometric measures, we extracted parameter estimates (betas) for the bilateral dlPFC and bilateral amygdala from clusters within each ROI in the contrasts with significant group differences. The extent of the clusters for extraction of betas was defined with an uncorrected voxelwise threshold of p<0.001.”

## Results

## **fMRI-ER task**

Our current analyses include predictor variables derived from the fMRI-ER data. The results of the fMRI-ER task have been reported previously by Seidel et al. (p. 4)^6^ and the relevant findings are described briefly below:

“Confirming that the fMRI-ER task elicited an expected activation pattern, exploratory analysis of the main effect of emotion induction (contrast: negative watch > neutral watch) over both groups revealed increased hemodynamic activity in the bilateral amygdala (left amygdala [-24 -6 16], k=215, t_peak_=9.94; right amygdala [22 -6 14], k=248, t_peak_=9.56). An independent-samples t-test showed significant differences between the groups within both a priori-specified ROIs (amygdala and dlPFC). Specifically, compared to HC, AN patients showed increased activity in the right amygdala ([36 2 −24], k=72, t_peak_=4.38) in response to passively viewing negative pictures as compared to passively viewing neutral ones. Furthermore, group differences in two clusters within the right dlPFC (right ventral dlPFC [38 40 2], k=53, t_peak_=4.24; right dorsal dlPFC [40 46 22], k=62, t_peak_=3.79) and two in the left dlPFC (left dorsal dlPFC [−36 44 1], k=112 t_peak_=4.1; left ventral dlPFC [−32 32 14], k=78, t_peak_=4.41) were found in the same contrast. An additional GLM with age as covariate confirmed the initial results.”

## fMRI-RX task: exploratory whole-brain analysis

Topographical and statistical details of an exploratory whole-brain analysis are provided in **SI Table 1**. Across all conditions, AN showed heightened activity in the right amygdala compared with HC. Negative pictures elicited increased activity in a cluster in the left fusiform gyrus/inferior temporal gyrus. The inverse contrast showed the left and right parahippocampal gyrus, the right posterior cingulate, the right subcallosal gyrus, and the left precentral gyrus to be particularly active in response to neutral pictures. There was no significant interaction between condition and group.

**SI Table 1**. Results of the exploratory whole-brain analysis of phase (2) fMRI-RX.

| **Contrast** | **Brain region** | **H** | **XYZ** | **K** | **Zmax** | **P** |
| --- | --- | --- | --- | --- | --- | --- |
| Main effects of group |  |  |  |  |  |  |
| AN > HC | amygdala | R | 26 -4 -16 | 11 | 5.43 | .01 |
| HC > AN | - | - | - | - | - | - |
| Main effects of condition |  |  |  |  |  |  |
| Negative > Neutral | fusiform gyrus | L | -42 -48 -18 | 20 | 4.98 | .005 |
| Neutral > Negative | parahippocampal gyrus  posterior cingulate  parahippocampal gyrus  precentral gyrus  subcallosal gyrus  posterior cingulate | L  R  R  L  R  R | -26 -42 -10  12 -56 20  30 -42 -8  -48 -18 48  8 12 -12  -10 -56 12 | 160  154  149  3  5  4 | 6.70  6.37  5.51  4.96  4.87  4.82 | .000  .000  .000  .026  .019  .022 |
| *Notes:* **H**=hemisphere, **XYZ**=MNI coordinates, **K**=cluster size (number of voxels), **Zmax**=peak z value, **P**=p-value of cluster, FWE-corrected. | | | | | | |

## **fMRI-RX task: arousal ratings**

| **SI Table 2.** Means (standard deviations) of arousal ratings during the fMRI-RX task. | | |
| --- | --- | --- |
| **Valence of stimuli** | **AN (Anorexia nervosa)** | **HC (Healthy controls)** |
| Neutral | -124.28 (55.03) | -103.64 (55.27) |
| Negative | 46.33 (79.02) | 59.28 (68.45) |
| *Notes:* The analogue rating scale ranged from ‘not aroused at all’ (-200) to ‘very aroused’ (+200). | | |

## fMRI-RX task: sustained effects of emotion regulation

Mean rated arousal during of phase (2) fMRI-RX did not differ between previous instructions (F(1, 70)=1.3, p=.29, η^2^=.02). There was no significant effect of group (F(1,70)=0.55, p=.46, η^2^=.008) or interaction between group and previous instruction (F(1, 70)=1.32, p=.25, η^2^=.02). Small volume corrected ROI analyses of (2) fMRI-RX data yielded no main effect of previous instruction (RX_negative_watch_ vs. RX_negative_distance_) and no significant interaction of previous instruction and group in any of the ROIs when applying the same significance levels (α=.05, voxelwise p-value p=.001) and minimum cluster sizes as in the main analyses (see 1.4).

## **Prediction of EMA data**

Due to technical data loss, the EMA groups differed in size (AN: n=36; HC: n=33), but comparable age was confirmed via an independent samples two-sided t-test (t=0.15, p=.86). To control for possibly confounding effects of compliance with the EMA protocol, individual percentage of completed questionnaires served as an additional predictor in a separate analysis. Direction and significance of effects did not change (see SI Table 3).

| **SI Table 3.** Non-standardized β-values of the EMA HLM analyses | | | | |
| --- | --- | --- | --- | --- |
|  | Phase (3) **EMA outcome variables** | | | |
|  |  | | **Controlled for compliance** | |
| **Predictors** | Negative affect | Tension | Negative affect | Tension |
| ***Level A***  Time of day  Company  Activity: leisure  Activity: eating  Activity: waiting  Activity: therapy  Activity: sleep  Activity: other | 0.78  5.05**  6.49*  -11.08**  -10.62**  -4.09  3.14  2.41 | 0.72  0.33  9.22**  -3.90  -5.49  5.23  11.38**  7.46* | 0.78  5.08**  6.49**  -11.08**  -10.61**  -4.11  3.14  2.43 | 0.72  0.34  9.22**  -3.89  -5.49  5.22  11.36**  7.46* |
| ***Level B***  Day of study  ***Level C***  Group  Phase (2) fMRI-RX amygdala  Phase (2) fMRI-RX amygdala * group  Compliance | -0.05  -33.66**  -2.71  1.66  - | 0.27  -13.61*  -7.67**  -2.54  - | -0.05  -31.25**  -3.11  1.53  -0.27 | 0.27  -13.24**  -7.75**  -2.56  -0.05 |
| *Notes.* Phase (3) EMA tension and negative affect predicted by group, mean phase (2) fMRI-RX amygdala activation, and their interaction (Level C). Phase (2) fMRI-RX amygdala activation was averaged over both hemispheres. Statistics include control variables on Level A (time of day, company, dummy-coded variables for activity, Level B (day of study) and Level C (compliance - two rightmost columns only). Negative affect, tension and all situational variables are given as measured by the EMA questionnaire; lower values indicate more tension and more negative affect. Group was coded -1=HC (healthy controls), 1=AN (Anorexia nervosa patients). *N*=69. *=significant at α≤.05; **=significant at α≤.01 | | | | |

# References

1. Munkres, J. Algorithms for the Assigment and Transportation Problems. *JSoc IndApplMath* **5**, 32–38 (1957).

2. Donnell, A. J., Pliskin, N., Holdnack, J., Axelrod, B. & Randolph, C. Rapidly-administered short forms of the Wechsler Adult Intelligence Scale—3rd edition. *Arch. Clin. Neuropsychol.* **22**, 917–924 (2007).

3. Waldmann, H.-C. Kurzformen des HAWIK-IV: Statistische Bewertung in verschiedenen Anwendungsszenarien. *Diagnostica* **54**, 202–210 (2008).

4. Lang, P., Bradley, M. & Cuthbert, B. International affective picture system (IAPS): Affective ratings of pictures and instruction manual. in *Technical Report A-8* (2008).

5. Wessa, M. *et al.* EmoPics: Subjektive und psychophysiologische Evaluation neuen Bildmaterials für die klinisch-bio-psychologische Forschung. *Z. Für Klin. Psychol. Psychother.* **39(Suppl. 1/11)**, (2010).

6. Seidel, M. *et al.* Processing and regulation of negative emotions in anorexia nervosa: An fMRI study. *NeuroImage Clin.* **18**, 1–8 (2018).

7. Seidel, M. *et al.* The real-life costs of emotion regulation in anorexia nervosa: a combined ecological momentary assessment and fMRI study. *Transl. Psychiatry* **8**, (2018).

8. Harris, P. A. *et al.* Research Electronic Data Capture (REDCap) - A metadata-driven methodology and workflow process for providing translational research informatics support. *J. Biomed. Inform.* **42**, 377–381 (2009).

9. Steyer, R., Schwenkmezger, P., Notz, P. & Eid, M. Testtheoretische Analysen des Mehrdimensionalen Befindlichkeitsfragebogen (MDBF). *Diagnostica* (1994).

10. Fichter, M. & Quadflieg, N. The structured interview for anorexic and bulimic disorders for DSM-IV and ICD-10 (SIAB-EX): reliability and validity. *Eur. Psychiatry* **16**, 38–48 (2001).

11. Maldjian, J. A., Laurienti, P. J., Kraft, R. A. & Burdette, J. H. An automated method for neuroanatomic and cytoarchitectonic atlas-based interrogation of fMRI data sets. *NeuroImage* **19**, 1233–1239 (2003).

12. Tzourio-Mazoyer, N. *et al.* Automated Anatomical Labeling of Activations in SPM Using a Macroscopic Anatomical Parcellation of the MNI MRI Single-Subject Brain. *NeuroImage* **15**, 273–289 (2002).

13. Ehrlich, S. *et al.* Associations of Cortical Thickness and Cognition in Patients With Schizophrenia and Healthy Controls. *Schizophr. Bull.* **38**, 1050–1062 (2012).

14. Ehrlich, S. *et al.* The COMT Val108/158Met polymorphism and medial temporal lobe volumetry in patients with schizophrenia and healthy adults. *NeuroImage* **53**, 992–1000 (2010).

15. Geisler, D. *et al.* Increased anterior cingulate cortex response precedes behavioural adaptation in anorexia nervosa. *Sci. Rep.* **7**, 42066 (2017).

16. Desikan, R. S. *et al.* An automated labeling system for subdividing the human cerebral cortex on MRI scans into gyral based regions of interest. *NeuroImage* **31**, 968–980 (2006).
